# Biochemical and Behavioral Consequences of Ethanol Intake in a Mouse Model of Metabolic Syndrome

**DOI:** 10.3390/ijms22020807

**Published:** 2021-01-15

**Authors:** Pablo Baliño, Ricard Romero-Cano, María Muriach

**Affiliations:** Unitat Predepartamental de Medicina, Universitat Jaume I, 12071 Castelló de la Plana, Spain; balino@uji.es (P.B.); romeror@uji.es (R.R.-C.)

**Keywords:** ethanol, diabetes, metabolic syndrome, oxidative stress, mice

## Abstract

Ethanol abuse is a common issue in individuals with sedentary lifestyles, unbalanced diets, and metabolic syndrome. Both ethanol abuse and metabolic syndrome have negative impacts on the central nervous system, with effects including cognitive impairment and brain oxidative status deterioration. The combined effects of ethanol abuse and metabolic syndrome at a central level have not yet been elucidated in detail. Thus, this work aims to determine the effects of ethanol intake on a mouse model of metabolic syndrome at the behavioral and biochemical levels. Seven-week-old male control (B6.V-Lep ob/+JRj) and leptin-deficient (metabolic syndrome) (B6.V-Lep ob/obJRj) mice were used in the study. Animals were divided into four groups: control, ethanol, obese, and obese–ethanol. Ethanol consumption was monitored for 6 weeks. Basal glycemia, insulin, and glucose overload tests were performed. To assess short- and long-term memory, an object recognition test was used. In order to assess oxidative status in mouse brain samples, antioxidant enzyme activity was analyzed with regard to glutathione peroxidase, glutathione reductase, glutathione, glutathione disulfide, lipid peroxidation products, and malondialdehyde. Ethanol intake modulated the insulin response and impaired the oxidative status in the ob mouse brain.

## 1. Introduction

According to the latest World Health Organization report on ethanol and health, more than 2.3 billion people are heavy ethanol consumers. Ethanol abuse has high social economic costs due to its elevated mortality and morbidity. In the European region alone, ethanol consumption is responsible for 10% of total deaths every year [1].

Ethanol abuse is related to obesity and an increased risk of metabolic syndrome (MS) and type 2 diabetes mellitus (T2D) [2]. MS occurs when different metabolic risk factors such as dyslipidemia, arterial hypertension, obesity, insulin resistance, and hyperinsulinism converge [3]. MS-elicited insulin resistance leads to impaired glucose homeostasis, which has been defined as a key mechanism involved in the pathogenesis of T2D [2,4,5,6,7]. Moreover, several factors such as diet, lifestyle, physical activity, mental health, and ethanol abuse interact with regard to the risk of suffering both pathologies [8,9,10]. In this respect, it has been demonstrated that high levels of ethanol consumption play a critical role in developing insulin resistance [11]. In fact, the World Health Organization has considered T2D to be a complication of ethanol abuse [1].

Chronic ethanol consumption threatens the oxidative defense of the organism and promotes the activation of different inflammatory processes which are related to the onset of MS [12], neurodegenerative damage [13,14], and beta cell death through activation of reactive oxygen species (ROS) [15,16]. Interestingly, some authors have described a paradoxical effect, and demonstrated how moderate ethanol consumption can modulate and improve insulin response in T2D [17,18]. Schaller and coworkers studied the effect of high ethanol consumption in T2D patients and observed an increase in the insulin response which modulates T2D [19,20,21,22,23,24,25,26,27,28]. One of the molecular mechanisms involves an ethanol-dependent increase in cAMP intracellular levels through a Ca^2+^-dependent mechanism. The intracellular calcium release elicited by ethanol thus boosts cellular insulin levels [29,30]. However, the mechanisms underlying the connection between T2D, MS, and ethanol abuse remain to be fully clarified.

On the other hand, it is widely known that ethanol metabolism is related to ROS production and mitochondrial injury, which are common features of acute and chronic ethanol exposure [31,32]. Via the dehydrogenase and microsomal ethanol oxidizing system, this ethanol-oxidative process generates NADH or NADP^+^. There is a subsequent increase in ROS levels, with the resultant oxidative stress representing an important threat to the oxidative system of the cell. Oxidative stress is also strongly correlated with prevalence of T2D and oxidative stress-induced tissue damage. Levels of different biomarkers such as 4-hydroxy-2-nonenal and malondialdehyde (MDA) as well as DNA base oxidation have been reported to be increased in the pancreatic tissue, plasma, and serum of T2D patients [32,33]. Moreover, it has been proven that the total antioxidant cellular defense is significantly diminished in T2D due to reduced levels of antioxidant metabolites such as catalase, superoxide dismutase, and the glutathione system, or nonenzymatic components such as vitamins C and E [32,34,35].

In this work, we aimed to evaluate the effect of chronic ethanol administration on a mouse model of MS, with a special focus on oxidative balance. Of the various animal models available for the study of MS, leptin-deficient mice (B6.Cg-Lep^Ob^) were chosen. These are known as ob mice and are homozygous for the leptin mutation, exhibiting obesity, hyperphagia, transient hyperglycemia, glucose intolerance, and elevated plasma insulin.

The effects of chronic ethanol administration on the central nervous system were determined through an object recognition test to ascertain cognitive impairment with regard to short- and long-term memory. The presence of cellular oxidative injury was determined using a battery of oxidative status parameters including the antioxidants glutathione reductase (GR), glutathione peroxidase (GPx), glutathione (GSH), glutathione disulfide (GSSG), and L-cysteine. Malondialdehyde (MDA) was used as a measure to study lipid peroxidation damage to macromolecules.

## 2. Results

### 2.1. Effects of Ethanol Intake with Regard to Weight, Glucose Overdose, Insulin, and Basal Glycemia

**Figure 1:**
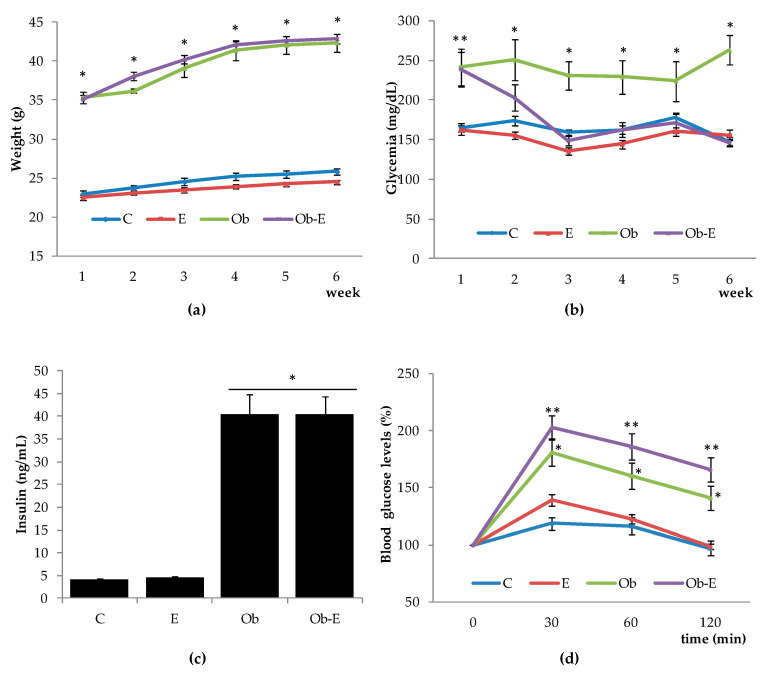
(**a**) Weekly animal weights (* *p* < 0.05 Ob-E and Ob vs. C and E); (**b**) weekly basal glycemia (* *p* < 0.05 vs. rest of groups, ** *p* < 0.05 vs. C and E); (**c**) blood insulin levels (* *p* < 0.05 vs. C and E); (**d**) blood glucose levels during the glucose overload test (%) (** *p* < 0.05 vs. all groups, * *p* < 0.05 Ob vs. C and E). C, wild-type control; E, wild-type ethanol; Ob, Ob control; Ob-E, Ob–ethanol.

In order to monitor weight changes elicited by chronic ethanol consumption, we monitored the weights of the mice over the course of the experiment. A two-way ANOVA showed significant differences between the wild-type (C, E) and MS groups (Ob, Ob-E) (*p* < 0.05, Figure 1a).

Glycemic status was monitored weekly. Significant differences were found by a two-way ANOVA between the wild-type (C, E) and MS mice groups (Ob, Ob-E) in week 1. The Ob-E group presented significantly reduced glycemia values when compared to the Ob control group from weeks 2–6 (*p* < 0.05, Figure 1b).

One-way ANOVA yielded significant differences in blood insulin concentrations between the wild-type (C, E) and MS mice groups (Ob, Ob-E) groups (*p* < 0.05, Figure 1c), with greater insulinemia found in the Ob and Ob-E groups.

Figure 1d depicts increased blood glucose levels (% t0) at 30, 60, and 120 min with respect to basal glycemia (time 0 min) over the course of the glucose overload test. A two-way ANOVA showed significant differences between the MS (Ob and Ob-E) and wild-type (C and E) mouse groups (Figure 1d, * *p* < 0.05). Thus, the Ob group presented greater increases in blood glucose levels as compared to the C and E groups. Moreover, the Ob-E group showed the greatest increases in blood glucose levels at 30, 60, and 120 min after glucose administration. These were significantly different when compared to the C, E, and Ob groups (Figure 1d, ** *p* < 0.05). 

### 2.2. Cognitive Effects of Ethanol Consumption

**Figure 2:**
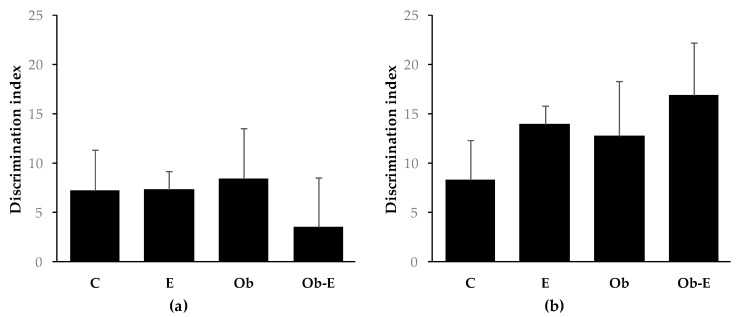
Effects of chronic ethanol exposure on wild-type and Ob mice in the object memory recognition task. Bars represent the mean ± SEM of the discrimination index evaluated at 1 h (short-term memory) (**a**) and 24 h (long-term memory) (**b**). Discrimation index is defined as DI = [(t_novel_ − t_familiar_)/(t_novel_ + t_familiar_) × 100%]. C, wild-type control; E, wild-type ethanol; Ob, Ob control; Ob-E, Ob–ethanol.

A novel object recognition test was used to assess short- (1 h) and long-term (24 h) memory alterations. No significant differences were observed between the wild-type (C, E) and Ob (Ob, Ob-E) groups (Figure 2).

### 2.3. Effects of Ethanol Intake on GR and GPx Activity

**Figure 3:**
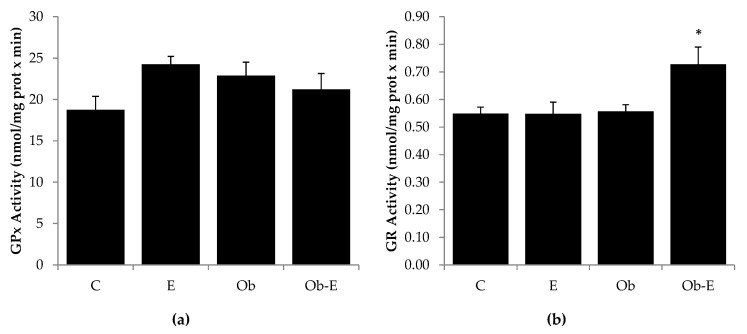
Effect of chronic ethanol exposure on enzymatic glutathione peroxidase (GPx) (**a**) and glutathione reductase (GR) activity (**b**) in the brain. Bars represent the mean ± SEM.* *p* < 0.05 vs. rest of groups. C, wild-type control; E, wild-type ethanol; Ob, Ob control; Ob-E, Ob–ethanol.

In this experiment, the enzymatic activity of brain GR and GPx was assessed. Figure 3 reflects the effects of ethanol administration on GPx and GR activity (Figure 3a,b, respectively). One-way ANOVA showed significant differences in GR enzymatic activity for the Ob-E group. Greater GR activity values were found for the Ob-E group as compared to the rest of the groups (* *p* < 0.05).

### 2.4. Effects of Ethanol Consumption on GSH, GSSG, GSH/GSSG Ratio, and L-Cysteine

**Figure 4:**
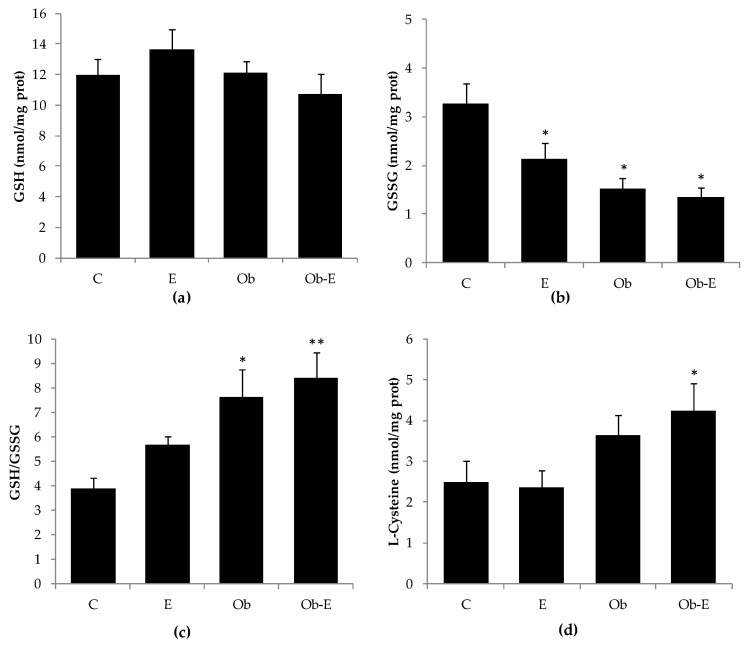
Effect of chronic ethanol exposure on brain glutathione (GSH) concentrations (**a**), glutathione disulfide (GSSG) concentrations (**b**) (* *p <* 0.05 vs. C), GSH/GSSG ratio (**c**) (* *p <* 0.05 vs. C, ** *p <* 0.05 vs. C and E), and L-cysteine values (**d**) (* *p <* 0.05 vs. C and E). Bars represent the mean ± SEM. C, wild-type control; E, wild-type ethanol; Ob, Ob control; Ob-E, Ob–ethanol.

No significant differences were observed in brain GSH levels after 6 weeks of ethanol consumption (Figure 4a). One-way ANOVA showed significant differences in brain glutathione disulfide (GSSG) concentrations. Post hoc comparisons indicated decreased GSSG concentrations in the E, Ob, and Ob-E groups as compared to the C group (Figure 4b; *p* < 0.05). With regard to the brain GSH/GSSG ratio, one-way ANOVA showed significant differences for the Ob versus C groups and for the Ob-E versus E and C groups (Figure 4c; *p* < 0.05). Post hoc comparisons demonstrated an increased GSH/GSSG ratio in Ob mice when compared to the C group, and in the Ob-E group versus the C and E groups.

We also measured L-cysteine concentrations in mouse brain samples. Post hoc comparisons indicated increased Ob-E brain L-cysteine concentrations as compared to the C group (Figure 4d, *p* < 0.05).

### 2.5. Effects of Ethanol Administration on MDA Levels

**Figure 5:**
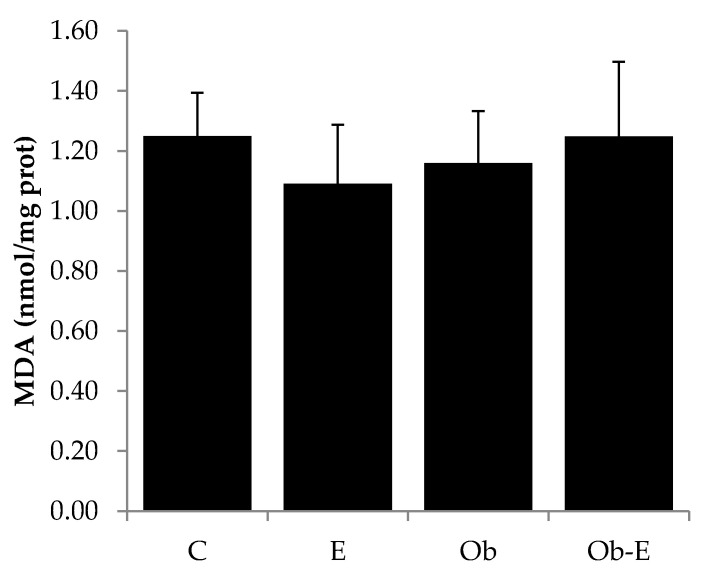
Effects of chronic ethanol administration on MDA concentrations. Bars represent the mean ± SEM. C, wild-type control; E, wild-type ethanol; Ob, Ob control; Ob-E, Ob–ethanol.

Oxidative damage to macromolecules was evaluated using the lipid peroxidation product MDA. One-way ANOVA showed no significant differences among groups (Figure 5).

## 3. Discussion

The results obtained in the present study showed that chronic ethanol intake improved fasting glycemic values but induced impaired glucose tolerance in MS model mice. Importantly, when measured over the time course of the experiment, no differences in weight were observed between the MS Ob mutant and Ob-E mice. Insulin levels in the Ob groups were significantly increased as compared to the wild-type, C, and E groups. As reported in the literature, this MS genetic model is defined (among other characteristics) by insulin resistance that leads to an altered glycemic response and hyperglycemia. Moreover, there was an increase in GR activity, GSH/GSSG ratio, and L-cysteine levels, indicating a compensatory mechanism due to the ethanol-elicited oxidative aggression after chronic ethanol intake.

### 3.1. MS Mice Model and Chronic Ethanol Consumption

In the present study, a MS genetic mouse model was used (B6.Cg-LepOb). Use of this model (known as Ob) is efficient because of the shortened time required for the development of MS as compared to the diet-induced MS model. In this regard, Ob mice are visually obese practically upon arrival and after 4 weeks of age the growing curve changes drastically and continues to increase even after 12 months of age. These mice have mild hyperglycemia, which transiently changes over time until sustained glucose blood levels of around 400 mg/dL are achieved. Interestingly, it is common to find peaking glucose elevations which result in β-cell failure. Moreover, this Ob model has elevated plasma cholesterol levels [36,37]. The ethanol intoxication model used in this study has been reported to achieve pharmacologically relevant blood ethanol concentrations [38,39].

Chronic ethanol consumption modulates glycemic levels and acute glycemic response. Insulin levels within the MS groups were increased when compared to those of wild-type animals (Figure 1c). It has been reported that chronic ethanol administration enhances cellular insulin levels [19,20,21,22]. One of the molecular mechanisms involved relates to an ethanol-dependent increase in intracellular cAMP levels through a Ca^2+^-dependent mechanism. The intracellular calcium release elicited by ethanol may play a prominent role as a mechanism involved in cellular insulin release [29,40] and the consequential observed hypoglycemia. Interestingly, as mentioned above, insulin levels within the MS groups (both Ob and Ob-E) were increased when compared to those of wild-type animals (Figure 1c) indicating that ethanol administration did not modify the impaired cellular insulin homeostasis of Ob mice. It is well known that β-cells compensate for the insulin resistance characteristic of this metabolic disorder, with an increase in secretory capacity or in beta-cell mass [36,37,40]. Moreover, insulin resistance appears to be a mechanism that overloads β-cells. This leads to malfunction and apoptosis [41], which result in the characteristic clinical manifestations of T2D: hypoinsulinemia and hyperglucagonemia [42,43]. In this respect, different mechanisms elicited by chronic ethanol administration have been described in the regulation of beta cell function [44]. On one hand, it has been described that chronic ethanol intake modulates glycemic levels through an increase in insulin release [19,20,21,22]. On the other hand, several authors have reported that the modulation of glycemic levels by ethanol is linked to its positive correlation with the development of abdominal obesity, which results in an increase in free fatty acid levels and lipogenesis. In this regard, mechanisms such as proinflammatory adipokine activity, glucokinase dysregulation, or GABA receptor inhibition underlie pancreatic beta-cell dysfunction and insulin release dysregulation [45,46,47,48]. While in our experimental conditions insulin levels were not modified by ethanol administration, we demonstrated that the acute glycemic response after a glucose overload worsened in the Ob-E group as compared to the Ob group (Figure 1d). Interestingly, fasting glycemic levels in chronic ethanol-treated MS animals were reduced to normal control values (Figure 1b). Moreover, it was also shown that ethanol administration in wild-type animals also reduced glycemia. It is known that ethanol metabolism inhibits gluconeogenesis and as a result, hepatic glucose production is initially upheld by glycogenolysis. Once the hepatic glycogen stores are depleted hypoglycemia may occur, explaining the late hypoglycemic effect of ethanol [49]. In addition, ethanol-induced hypoglycemia may be associated with blunted nocturnal growth hormone response, impaired counterregulatory response, and impaired ketogenesis [50]. Type 2 diabetes is a progressive disease which typically starts with a gradual loss of glycemic control after meals followed by the development of fasting hyperglycemia [51,52]. Our results indicate that ethanol causes a deterioration in postprandial glucose (Figure 1d) before fasting glucose (Figure 1b) in Ob mice. This supports the idea of a time-dependent effect of ethanol on cellular glycemic homeostasis. Preliminary data obtained in our laboratory demonstrated that ethanol administration to MS animals for 6 months aggravated the glucose overload response and reduced insulin release in Ob-E mice. Further studies are necessary to demonstrate whether this reduction is due to the overload, malfunction, or apoptosis of β-cells characteristic of T2D.

### 3.2. Chronic Ethanol Consumption and MS Modulate Oxidative Status in the Brain of Mice

Postprandial hyperglycemia may induce oxidative stress [53], and central ethanol metabolism plays a critical role in the disruption and loss of the cellular oxidative balance [54]. There is a large body of evidence indicating that at a central level, ethanol is oxidized through a catalase–H_2_O_2_ system. This oxidative reaction results in the production of ethanol’s first oxidative metabolite, acetaldehyde, which is highly reactive and responsible for cell injury [55,56,57,58]. In our experimental conditions, we observed that ethanol administration was able to increase brain GR activity as well as the GSH/GSSG ratio and L-cysteine concentrations in Ob-E mice (Figure 4). These results support the hypothesis of a compensatory mechanism after an oxidative insult based on a temporary cellular increase in antioxidant defense to restore the original homeostatic conditions [59,60]. Due to the formation of reactive species the glutathione system is activated to reestablish cell redox balance. When a harmful oxidant agent is present in the cell, GPx is activated, reducing the reactive species and oxidizing GSH to its disulfide form (GSSG). Thus, GSSG is reduced to GSH through GR enzyme activity, restoring initial GSH concentrations. In this process, GSH and its disulfide form GSSG remain in constant enzyme substrate turnover. Our data showed an increase in GR activity which was not accompanied an enhanced GPx activity, thus justifying the reduced GSSG concentration and increased GSH/GSSG ratio. Other studies have found an increase in the enzymatic activity of GPx after ethanol administration [61,62]. This discrepancy may be due to the fact that these studies explored the acute effects of ethanol administration. There is a large body of evidence indicating that in the brain, acetaldehyde is directly formed via the catalase–H_2_O_2_ system [54]. In this respect it may be hypothesized that after 6 weeks of ethanol administration GPx activity does not increase because its substrate, H_2_O_2_, appears to be reduced by the catalase system. Moreover, we observed an increase in brain L-cysteine concentrations in the Ob-E group (Figure 4). The increase in levels of this antioxidant supports the hypothesis of a compensatory mechanism [63]. Thus, in animals with MS, chronic ethanol administration modulates GSH metabolism, resulting in elevated concentrations of at least one of its forming peptides.

With respect to brain MDA concentrations, lipid peroxidation is a process which involves the oxidation of polyunsaturated fatty acids present in the biological membranes. The products of this lipid peroxidation (such as MDA) yield several DNA adducts, are extremely mutagenic, and cause protein damage [64,65,66]. It has been demonstrated that lipid peroxidation can be increased by ethanol administration, which is accompanied by diminished cell antioxidant defense [67,68]. However, in our experimental conditions this ethanol-induced peroxidative damage appeared to be offset by the increased response of the cellular antioxidant system (Figure 3 and Figure 4).

### 3.3. Behavioral Effects of Chronic Ethanol Consumption in MS Mice

At the behavioral level, 6 weeks of chronic ethanol administration modulated different processes involved in glucose homeostasis, acute glycemic response, and brain oxidative status, but did not have behavioral effects in our model of MS. In this respect, different authors have found behavioral alterations within the same time-course of ethanol administration. However, the experimental procedure used to obtain the effects of ethanol intoxication was based on a different diet (Lieber-de-Carli) [67]. Thus, in our experimental conditions, short- and long-term recognition memory processes were not affected. In fact, this ethanol administration model was proven to produce behavioral impairment after 5 months of ethanol consumption [38,39].

## 4. Materials and Methods

### 4.1. Animals

A total of 96 male C57BL6 and B6.Cg-Lep^Ob^ (Janvier Labs, Le Genest-Saint-Isle, France) mice were used in this study. The animals, which were 4 weeks of age upon arrival, were housed five per cage in an acclimated quarantine room in which they remained for a week. After this period, mice were moved into the colony room for a week before ethanol exposure. Mice were 6 weeks of age when ethanol was introduced for 6 weeks. The colony room was maintained at a temperature of 21 ± 1 °C, and controlled under a 12-h light/dark cycle (lights on at 8:00 a.m.). Food and water were provided ad libitum throughout the study. All experimental procedures complied with the European Community Council Directive (2010/63/EU) and were approved by the Animal Health department of Generalitat Valenciana (project code: 2019/VSC/PEA/0053, approved date: 11 March 2019).

### 4.2. Drugs and Chemicals

Ethanol (Panreac, Barcelona, Spain) was diluted to 12% (*v*/*v*) in water and administered.

To prepare working solutions and assay reactants potassium hydrogenphophate, potassium dihydrogenphosphate, glutathione oxidized, glutathione reduced, L-cysteine, 1-fluoro-2,4-dinitrobencene, iodoacetic acid, perclhoric acid, glacial acetic acid, sodium acetate, ethylenediaminetetraacetic acid (EDTA), sodium azide (NaN_3_), GSH disulfide reductase (GR), hydrogen peroxide 30%, 5,5′-dithiobis(2-nitrobenzoic acid) (DTNB), sodium hydrogenphophate, sodium dihydrogenphosphate, 2-thiobarbituric acid and 1,1,3,3-tetramethoxypropane were purchased from Sigma-Aldrich (Darmstadt, Germany). Methanol and NADPH were purchased from Scharlab (Barcelona, Spain) and Panreac (Barcelona, Spain), respectively. High-purity water was obtained from a Millipore System (Merk Millipore, Burlington, MA, USA).

### 4.3. Experimental Procedure

Animals were divided into four groups (*n* = 10–12 per group): wild-type control (C), wild-type ethanol (E), MS control (Ob), and MS ethanol (Ob-E). The E and Ob-E groups were exposed to ethanol (12% *v*/*v*) in drinking water for 6 weeks. To avoid treatment rejection, the ethanol concentration was scaled over a 2-week habituation period (0%, 2%, 4%, 6%, 8%, 10%, and 12%, every 48 h). The blood ethanol levels achieved in the E and Ob-E groups were 108.5 ± 18.0 mg/dL and 95.6 ± 22.6 mg/dL, respectively.

Weight, glycemia, and liquid consumption values were determined once a week, 2 h after lights were turned on. Animals were moved from their home cages to the procedure room 30 min before the start of each experiment, allowing them to acclimatize to the environmental conditions. Finally, animals were sacrificed using cervical dislocation. Blood samples and brain samples were frozen to −80 °C for further analysis. Prior to freezing, brain samples were homogenized in prechilled 0.2 M potassium phosphate buffer, pH 7.

#### 4.3.1. Glycemia, Insulinemia, and Glucose Overload Tests

Glycemia was measured weekly through the collection of 1 µL of blood from the mouse tail. Glucose levels were then measured using an ACCU-CHECK glucometer (Roche Diagnostics, Rotkreuz, Switzerland). Blood insulin levels were assayed using an Elisa Kit (Merk, Darmstad, Germany). For the glucose overload test, fasting mice (12 h) were treated with glucose (2 g/kg i.p.), and blood glucose levels were determined after 30, 60, and 120 min. Glycemic values at 30, 60, and 120 min were represented as a % of increase versus time 0.

#### 4.3.2. Memory and Learning Test

Short- and long-term memory, as well as the behavioral effects of ethanol, were evaluated using the object recognition test. Briefly, a 40-cm diameter cylinder with sawdust on the floor was set down in a testing room with faint light. Two red plastic blocks were placed inside the cylinder and a mouse was allowed to inspect objects for 5 min (training session). To measure short-term memory, one of the blocks was changed to a round yellow block. All the objects were heavy enough to prevent displacement. One hour after the end of the training session animals were placed in the middle of the cylinder and allowed to explore the objects for 5 min to evaluate short-term memory. The experiment was repeated 24 h after the end of the training session to evaluate long-term memory. The orientation of the animal’s snout toward the object within a range of 2 cm or less from the object was defined as object exploration. Sitting on the object or running around it was not recorded as exploration. Objects were washed with ethanol after each individual trail to equate olfactory cues. The discrimination index, calculated as [DI = (*t*_novel_ − *t*_familiar_)/(*t*_novel_ + *t*_familiar_) × 100%], was used as a basic measure in the object recognition test [38].

The oxidative status of brain samples was evaluated as follows:

#### 4.3.3. Antioxidant Defenses

Glutathione (L-Glutamyl-L-cysteinyl-glycine) is a tripeptide with multiple cellular functions. Glutathione is the main antioxidant system in the cell and a detoxification agent for ROS and reactive nitrogen species. There are two forms of glutathione: the oxidized form (GSSG) and the reduced form (GSH). The GSH/GSSG redox couple is a cosubstrate for glutathione peroxidase and reductase enzymes (GPx and GR) which act as a vital cellular redox buffer modulating different sensitive biochemical and biophysical processes. L-cysteine is a glutathione biosynthesis precursor used as an oxidative stress biomarker [69].

##### GSH, GSSG, and L-Cysteine

GSH, GSSG, and L-cysteine concentrations were chromatographically quantified using Reed’s method [70]. This method derivatizes the amino groups with 1-fluoro-2,4-dinitrobencene (Sanger reactant) after blocking the free thiol groups with iodoacetic acid. Briefly, samples (180 µL with 20 µL perclhoric acid 20%) were centrifuged, and then iodoacetid acid (40 µL, 0.1 M) was added. After adjusting to pH 9 and with obscurity and ice-cold incubation for 30 min, Sanger reactant (200 µL) was added. Samples were then incubated overnight at 4 °C. Samples were centrifuged and filtered through a 22-µm nylon filter prior to the chromatographic analysis. The high-performance liquid chromatography (HPLC) working solutions were methanol:water (4:1, *v*/*v*) (mobile phase A) and methanol:sodium acetate buffer 3.45 M pH 5.3 (64:36, *v*/*v*) (mobile phase B). Separation was carried out using a Kromasyl-NH2 250 × 4.6 mm 5 μm column (Waters Corporation, Mildford, MA, USA), and the UV-Vis detector was set at 365 nm. The separation gradient was 20% B from initial time to 10 min, which was then increased linearly to 80% B at 45 min at a 1 mL/min flow.

##### Glutathione Peroxidase Activity (GPx)

GPx was measured by monitoring the disappearance of NADPH at 340 nm [71]. Briefly, 50-μL samples were mixed with potassium phosphate buffer 0.1 M pH 7.0 (550 µL) containing ethylenediaminetetraacetic acid (EDTA), NaN_3_ (both 1 mM), and 100 µL of the following solutions: GSH disulfide reductase (0.24 U/mL), reduced glutathione (1 mM), and NADPH (0.15 mM). After incubation (3 min at 37 °C), a reaction was started by the addition of hydrogen peroxide 1.5 mM (100 µL).

##### Glutathione Reductase Activity (GR)

GR was determined spectrophotometrically using Smith’s proposed method [72]. Briefly, when the GR catalyzed reduction of GSSG to GSH was produced in presence of 5,5′-dithiobis(2-nitrobenzoic acid) (DTNB), 2-nitrobenzoic acid was formed as a subproduct. Formation was monitored at 412 nm. The GSSG reduction was started by adding 25 µL of brain sample to a solution containing DTNB 3 mM prepared in 10 mM sodium phosphate buffer, 2 mM NADPH, and 10 mM EDTA in 0.2 M pH 7.5 sodium phosphate buffer.

The oxidative damage to macromolecules was evaluated as follows:

Lipid peroxidation or reaction of oxygen with unsaturated lipids produces a wide variety of oxidation products. One of the main primary products and a biomarker for lipid peroxidation is MDA, which appears to be the most mutagenic and cell-toxic agent [73].

#### 4.3.4. Lipid Peroxidation

MDA concentrations were determined chromatographically using an HPLC system equipped with a fluorescence detector (set to 527 nm for excitation and 532 nm for emission). Sample preparation consisted of mixing samples (100 µL) with 0.75 mL of thiobarbituric acid 0.37% and perchloric acid 6.4% (2:1, *v*/*v*), heating to 95 °C for an hour. Prior to injection in the HPLC system, the pH was adjusted to 6 and precipitates were removed by centrifugation (10,000 rpm, 1 min). Separation was carried out in a C18 250 × 4.6 mm 5 µm column (Scharlab, Barcelona, Spain) and flow was set at 1 mL/min in an isocratic separation. Mobile phase consisted of a 50-mM potassium phosphate buffer (pH 6.0):methanol (58:42, *v*/*v*). Standard solutions were prepared daily from 1,1,3,3-tetramethoxypropane. This method was first proposed by Richard [74] and later modified by Romero and cols. [75].

#### 4.3.5. Protein Content

Protein levels were determined from brain lysates in accordance with the method described by Bradford [76] (ThermoFisher Scientific, Waltham, MA, USA).

#### 4.3.6. Statistical Analysis

Statistical analyses were carried out using SPSS software (IBM SPSS Statistics for Windows, Version 25.0 IBM Corp, Armonk, NY, USA). Results were presented as mean values ± SE. Comparisons between groups were made by one-way ANOVA. The analysis of variance of the obtained data was performed by the Levene test, using the LSD test as a post hoc test when the data showed homogeneity in their variances (*p* < 0.05), or a Dunnet T3 test when variances differed. Statistical significance differences were set at the *p* < 0.05 level.

## 5. Conclusions

This body of evidence supports the hypothesis that ethanol is a modulating agent of glycemic response in MS mice. This modulation was accompanied by a dysregulation of the brain oxidative status but did not affect short- and long-term memory, indicating moderate effects of ethanol consumption on behavior. However, further studies are required to elucidate the particular mechanisms by which ethanol might modulate the transition from MS to T2D.

## Data Availability

Data sharing not applicable.

## Abbreviations

cAMP	Cyclic adenosine monophosphate
DI	Discrimination index
DTNB	5,5′-dithiobis(2-nitrobenzoic acid)
EDTA	Ethylenediaminetetraacetic acid
GABA	Γ-aminobutyric acid
GPx	Glutathione peroxidase activity
GR	Glutathione reductase activity
GSH	Glutathione
GSSG	Glutathione disulfide
HPLC	High-performance liquid chromatography
L-Cys	L-cysteine
MDA	Malondialdehyde
MS	Metabolic syndrome
NADPH	Nicotinamide adenine dinucleotide phosphate
T2D	Type 2 diabetes
WHO	World Health Organization
